# Comprehensive Analyses of Four *PtoNF-YC* Genes from *Populus tomentosa* and Impacts on Flowering Timing

**DOI:** 10.3390/ijms23063116

**Published:** 2022-03-14

**Authors:** Juan Li, Kai Gao, Xiaoyu Yang, Bin Guo, Yinxuan Xue, Deyu Miao, Sai Huang, Xinmin An

**Affiliations:** 1 Beijing Advanced Innovation Center for Tree Breeding by Molecular Design, National Engineering Research Center of Tree Breeding and Ecological Restoration, College of Biological Sciences and Technology, Beijing Forestry University, Beijing 100083, China; 15735175984@163.com (J.L.); gaokai@caf.ac.cn (K.G.); xiaomoziyu@126.com (X.Y.); guobin531188058@163.com (B.G.); xueyinxuan@bjfu.edu.cn (Y.X.); miaodeyu@163.com (D.M.); huangsai@bjfu.edu.cn (S.H.); 2 Key Laboratory of State Forestry and Grassland Administration on Tropical Forestry Research, Research Institute of Tropical Forestry, Chinese Academy of Forestry, Guangzhou 510520, China; 3 Research Institute of Subtropical Forestry, Chinese Academy of Forestry, Hangzhou 311400, China; 4 Shanxi Academy of Forestry and Grassland Sciences, Taiyuan 030012, China

**Keywords:** flowering time, genome-wide analysis, NF-YC, *Populus tomentosa*, transgenic plant

## Abstract

Flowering is an important link in the life process of angiosperms, and it is also an important sign of the transformation of plants from vegetative to reproductive growth. Although the flowering regulation network of Arabidopsis is well-understood, there has been little research on the molecular mechanisms of perennial woody plant flower development regulation. *Populus tomentosa* is a unique Chinese poplar species with fast growth, strong ecological adaptability, and a long lifecycle. However, it has a long juvenile phase, which seriously affects its breeding process. Nuclear factor-Y (NF-Y) is an important type of transcription factor involved in the regulation of plant flowering. However, there are few reports on *PtoNF-Y* gene flowering regulation, and the members of the PtNF-YC subfamily are unknown. In this study, four key genes were cloned and analyzed for sequence characteristics, gene structure, genetic evolution, expression patterns, and subcellular localization. The plant expression vector was further constructed, and transgenic Arabidopsis and *P. tomentosa* plants were obtained through genetic transformation and a series of molecular tests. The flowering time and other growth characteristics were analyzed. Finally, the expression level of flowering genes was detected by quantitative PCR, the interaction between PtoNF-YC and PtoCOL proteins was measured using the yeast two-hybrid system to further explain the flowering regulation mechanism, and the molecular mechanisms by which *PtNF-YC6* and *PtNF-YC8* regulate poplar flowering were discussed. These results lay the foundation for elucidating the molecular regulation mechanism of *PtoNF-YC* in flowering and furthering the molecular design and breeding of poplar, while providing a reference for other flowering woody plants.

## 1. Introduction

The transition from vegetative growth to reproductive growth is an important step in the plant lifecycle, and flowering is a key sign of this transition [1]. Compared with annual plants, most woody plants need a longer juvenile period before entering the flowering period, and many traits can only be expressed after reaching maturity. However, economic forests and timber forests have the problems of long a juvenile period and late flowering, which seriously limit the improvement of their economic benefits and production development, as well as their breeding process [2,3,4]. Therefore, promoting early flowering, shortening the juvenile period, and accelerating the breeding cycle of trees are of great significance to the development of forestry science and the study of the molecular mechanisms of plant sexual reproduction.

Nuclear factor-Y transcription factors also called the heme activator protein (HAP) or CCAAT-binding transcription factor, are ubiquitous in animals, plants, and other eukaryotes [5,6,7]. NF-Y complexes bind to the CCAAT motif in the promoters of many genes [8,9]. NF-Y transcription factors are a heterotrimeric complex composed of three subunits: NF-YA, NF-YB, and NF-YC. Among them, NF-YB and NF-YC form dimers in the cytoplasm, and further combine with the NF-YA protein in the nucleus to form heterotrimers [10]. In yeast and mammals, each NF-Y subunit is encoded by one gene [11]. By contrast, each NF-Y subfamily in plants consists of multiple members. At present, NF-Y family members have been identified in many species, including *Arabidopsis* [12], rice [13], maize [14], tomato [15], poplar [16], and *Pinus tabuliformis* [17]. Some species members expanded, resulting in functional redundancy and functional differences, while also helping to form a transcription factor network to regulate plant growth and development [18,19].

NF-Y is involved in multiple growth and development processes of plants, including embryogenesis, seed germination, flowering, fruit ripening, and other growth processes [15,20,21,22,23,24]. Furthermore, NF-Y plays an important role in responding to abiotic stress, such as drought, high salt, and low temperature [25,26,27]. Notably, NF-Y plays an important role in the process of flowering regulation, especially the NF-YC subfamily, which participates in flowering regulation in different ways. For example, in the photoperiod-dependent flowering pathway, Arabidopsis AtNF-YB2 and AtNF-YB3 bind to AtNF-YC3, AtNF-YC4, and AtNF-YC9, and the heterodimers further interact with CONSTANS to induce *FT* expression [22]. In the aging-regulated flowering pathway, *CmNF-YB8* regulates flowering time by regulating the expression of the *cmo-MIR156* gene in the senescence pathway [23]. In addition, the NF-Y complex can also act as an epigenetic regulator in the gibberellic acid pathway [28]. However, little is known about the poplar *NF-Y* gene in flowering. In recent years, studies have shown that overexpression of poplar *PtNF-YB2* in *Arabidopsis* and tomato can induce earlier flowering [3], and overexpression of *PtNF-YA9* can delay flowering in Arabidopsis [29]. However, research on the flowering of poplar NF-YC is lacking.

*Populus tomentosa* is a unique Chinese poplar species with rapid growth, strong ecological adaptability, and a long lifecycle. It plays an important role in forestry economy, ecological construction, and urban greening [2,4,16]. However, *P. tomentosa* has a long juvenile phase, which seriously affects its breeding process. Although NF-Y is an important transcription factor involved in regulating plant flowering, there are few reports on *NF-Y* gene flowering regulation in poplar, and the members of the PtNF-YC subfamily are unknown.

In this study, four *PtoNF-YC* genes were cloned from *P. tomentosa*. We confirmed the function of the *PtoNF-Y6* and *PtoNF-Y8* in regulating flowering timing using transgenic *Arabidopsis* and *P. tomentosa*. Based on the result, we proposed a potential molecular mechanism model of *PtoNF-Y6* and *PtoNF-Y8* in flowering regulation. The results of this study lay the foundation for elucidating the molecular regulation mechanism of *PtNF-YC* in flowering, and provide a reference for research on flowering regulation in other woody plants.

## 2. Materials and Methods

### 2.1. Plant Material and Growth Conditions

In this study, roots, stems, leaves, leaf buds, flower buds, and flowers of *P. tomentosa* were collected as previously described [16]. For poplar genetic transformation, 2-month-old *P. tomentosa* (TC1521, female clone) seedlings were used as described by Li [30]. All plants were grown in the Beijing Forestry University greenhouse (Beijing, China) and maintained at 23 ± 1 °C and 60–70% humidity. For *Arabidopsis* transformation, wild-type (WT) ecotype Columbia (*Col*) was used as the experimental material. It was grown in long-day conditions (16 h light/8 h dark) at 20–22°C.

### 2.2. Cloning and Sequence Analysis of the Four PtoNF-YC Genes

The total RNA was extracted from tissue culture seedlings of *P. tomentosa* using the TRIzol Total RNA Extraction Kit (Promega, Madison, WI, USA). RNA quality was measured using a NanoDrop 2000 spectrophotometer (Implen, Inc., Westlake Village, CA, USA). RQ1 DNase was used to remove genomic DNA, and the Reverse Transcription System (Promega, Madison, WI, USA) was used to synthesize cDNA according to the manufacturer’s instructions. Using the *Arabidopsis* NF-YC protein sequences as the query sequences, those with high similarity to *Arabidopsis* NF-YC were obtained from the Phytozome v12.1 database using BLAST. All cloning primers were designed using the Primer Premier 5.0 software [31], and are presented in Table S1. The PCR program was as follows: 95 °C for 3 min, 35 cycles of 30 s each at 95 °C, 30 s at 60 °C, and 1 min at 72 °C, and a final elongation step of 72 °C for 5 min. The PCR products were detected by 1.5% agarose gel and the target bands were purified as previously described [32]. Then, the purified band was cloned into the pGEM-T Easy Vector (Promega) for sequencing. The physicochemical parameters of the PtoNF-YC proteins were predicted by Expasy (http://cn.expasy.org/, accessed on 3 January 2022) [33]. Multiple alignment analysis of the sequences was performed with ClustalX2.1 and GenDoc software [34]. The phylogenetic tree was generated by MEGA7.0 using the neighbor-joining method based on the NF-Y protein sequences [35].

### 2.3. PtoNF-YC Expressing Vector Construction and Subcellular Localization Assay

To express the PtoNF-YC protein, we ligated the four cloned *PtoNF-YC5*, *PtoNF-YC6*, *PtoNF-YC7*, and *PtoNF-YC8* genes into pSuper1300-GFP with the *eGFP* gene to produce pSuper1300-GFP-PtoNF-YC5, pSuper1300-GFP-PtoNF-YC6, pSuper1300-GFP-PtoNF-YC7, and pSuper1300-GFP-PtoNF-YC8 constructs, respectively. The constructed vectors were verified by PCR and double enzymatic digestion, and transferred into the *Agrobacterium* expression strain GV3101. For subcellular localization analysis, *Agrobacterium* containing the expression vector was cultured to an optical density value of 1.0, harvested, resuspended in agroinfiltration buffer (150 μM acetosyringone, 10 mM MgCl_2_, and 10 mM MES), and injected using a syringe into tobacco leaves. Three days after the injection, a Leica TCS SP8 confocal microscope was used to observe the results.

### 2.4. Analysis of the Interaction Mode of PtoNF-YCs and PtoCOL

We performed yeast two-hybrid (Y2H) experiments using the Gal4-based two-hybrid system according to the manufacturer’s instructions (Clontech Laboratories, San Jose, CA, USA). The open reading frames (ORFs) of poplar *PtoNF-YC6* and *PtoNF-YC8* were inserted into the bait vector pGBKT7, and finally pGBKT7-PtoNF-YC6 and pGBKT7-PtoNF-YC8 were formed as bait, respectively. The PtoNF-COL1 and PtoNF-COL2 ORFs were cloned into the vector pGADT7. The primers used are listed in Table S1. Then, the pGBKT7-PtNF-YC6/8 constructs and pGADT7-PtNFCOL1 and pGADT7-PtNF-COL2 were respectively transformed into the yeast strain Y2HGold in pairs. Then pGBKT7-53 and pGBKT7-Lam were used as positive and negative control plasmids, respectively. Finally, positive colonies were selected using synthetic defined medium without Trp-Leu-His-Ade (SD-Trp-Leu-His-Ade medium).

### 2.5. Arabidopsis and Poplar Transformation

For the transformation of *Arabidopsis*, *PtoNF-Y6,* and *PtoNF-Y8* overexpression constructs were introduced into *Col* using the floral dip method with *Agrobacterium* GV3101 strain [36]. The seeds of the *PtoNF-YC6* and *PtoNF-YC8* transgenic lines were harvested separately and sown again, and finally the homozygous transgenic lines were used for further research. For poplar transformation, we used 2-month-old *P. tomentosa* TC1521 tissue culture seedlings as explant material, and further transformed explants through *Agrobacterium*-mediated leaf disc [30].

### 2.6. Extraction of RNA and Quantitative PCR Analysis

Roots, stems, leaves, leaf bud, flower bud, and flower were obtained for tissue-specific expression pattern analysis. The *PtoNF-YCs* overexpressing plants and wild-type plants were used to detect the expression level of *PtoNF-YC6* and *PtoNF-YC8* in poplar and *Arabidopsis*. All samples were immediately frozen in liquid nitrogen and then stored at −80 °C until further use. Next, the total RNA of different tissues and transgenic plants were extracted and reverse transcribed as described above. The quantitative PCR (qPCR) primers were designed using Primer Premier 5.0, and the *PtACTIN* gene was used as the internal control [2,37,38]. All primers are listed in Table S1. The expression levels of the target genes were detected using SYBR^®^ Premix ExTaq™ (TaKaRa Bio, Shiga, Japan) on an ABI PRISM 7500 Fast Real-time PCR System (Applied Biosystems, Foster City, CA, USA). The PCR program used was previously described [16]. Data were analyzed using the 2^−ΔΔCT^ method [39].

### 2.7. Phenotypic Analysis and Biomass Measurements

The phenotypes of transgenic *Arabidopsis* and *P. tomentosa* plants were recorded, including total height, stem diameter, flowering time, and floral organ variation.

### 2.8. Statistical Analyses

In the study, three wild-type and transgenic plant lines were selected (except the overexpressing *PtoNF-YC8 P. tomentosa* plants were two lines), with three plants per line, and each experiment was repeated five times. All presented data are from the mean of three experiments. The data are presented as the mean ± standard error of the mean and were analyzed using the SPSS 19.0 software (IBM Inc., Armonk, NY, USA). The error bars were calculated according to Tukey’s multiple range test, and with * (*p* < 0.05), ** (*p* < 0.01) being used to indicate statistically significant effects.

## 3. Results

### 3.1. Cloning and Sequence Analysis of the Four PtoNF-YC Genes

Previous research by our group showed that the four PtNF-YC5/6/7/8 proteins form an independent subfamily with the *Arabidopsis* flowering-related proteins AtNF-YC3, AtNF-YC4, and AtNF-YC9 (Figure S1). In addition, the expression patterns of flower bud development indicated that *PtoNF-YC5* and *PtoNF-YC6* have higher expression levels in the dormancy and sporulation phases, while *PtoNF-YC7* and *PtoNF-YC8* have higher expression levels in the early stages of flower bud development [16]. Thus, we speculated that PtoNF-YC5/6/7/8 members play an important role in flower development.

To obtain the four *NF-YC* genes of *P. tomentosa*, we used the cDNA of *P. tomentosa* TC1521 as a template to perform PCR, and finally obtained four fragments of 753 bp, 711 bp, 681 bp, and 777 bp, respectively. The PCR product was purified and ligated into the pGEM-T Easy Vector for sequencing. Finally, the four genes that were sequenced correctly were named PtoNF-YC5, PtoNF-YC6, PtoNF-YC7, and PtoNF-YC8 (Figure S2).

**Figure 1:**
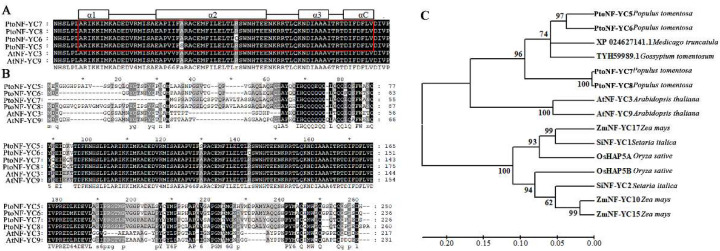
Comparison of the four PtoNF-YC proteins with NF-YC protein sequences from other species. (**A**) Comparison of conserved sequence regions of *P. tomentosa* PtoNF-YC5/6/7/8 and *Arabidopsis* homologous protein. (**B**) Comparison of *P. tomentosa* PtoNF-YC5/6/7/8 and *Arabidopsis* homologous protein sequence. (**C**) Phylogenetic analysis of PtoNF-YC5/6/7/8 homologous protein sequence in *P. tomentosa*.

To further analyze the evolutionary relationship between the PtoNF-YC5/6/7/8 protein and NF-YC proteins in other species, a comprehensive phylogenetic tree was constructed using NF-YC proteins from a variety of monocot and dicot plants. The results showed that these proteins were divided into two major branches. Among them, the NF-YC protein of monocots *Zea mays*, *Setaria italica*, and *Oryza sativa* formed one branch, while dicots formed another branch. The dicot branch was further divided into two sub-branches: *Arabidopsis thaliana* formed one sub-branch, and *Populus*, *Gossypium*, *Medicago*, and other plants formed the other sub-branch. PtoNF-YC5 protein showed the closest relationship with PtoNF-YC6, while PtoNF-YC7 had the closest relationship with PtoNF-YC8. This also showed that the functions of the two pairs of members of PtoNF-YC5/6 and PtoNF-YC7/8 are similar or redundant (Figure 1C). The conserved domain analysis showed that the four proteins PtoNF-YC5/6/7/8 all have a core histone sequence, indicating that they belong to the PtoNF-YC family of transcription factors (Figure 1A,B).

### 3.2. Tissue-Specific Expression and Subcellular Localization Analysis of the Four PtoNF-YC Genes

**Figure 2:**
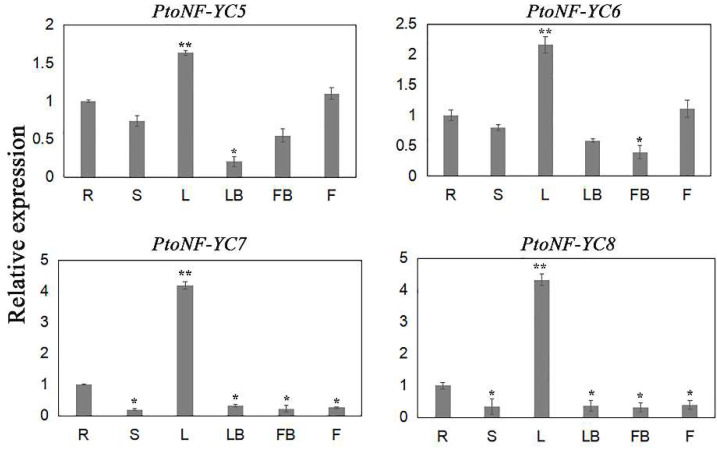
Tissue-specific expression patterns of *PtoNF-YC5*/*6*/*7*/*8* in *P. tomentosa*. R, S, L, LB, FB, and F represent roots, stems, leaves, leaf buds, flower buds, and flowers, respectively. The data was presented as mean ± SE (n = 3 independent replicates), * (*p* < 0.05) and ** (*p* < 0.01) indicate significant differences.

To further investigate the potential functions of *PtoNF-YC* genes in different tissues and organs of *P. tomentosa*, we analyzed the expression patterns of *PtoNF-YC5*/*6*/*7*/*8* using qPCR. The four *PtoNF-YC* genes were expressed in different tissues in *P. tomentosa*, with different expression levels. In the vegetative growth stage, expression levels were the highest in leaves. In the reproductive growth stage, the expression levels of *PtoNF-YC5* and *PtoNF-YC6* in flower buds and flower were higher than those of *PtoNF-YC7* and *PtoNF-YC8*. Moreover, all four genes showed the highest expression in leaves, indicating that they may be redundant (Figure 2).

**Figure 3:**
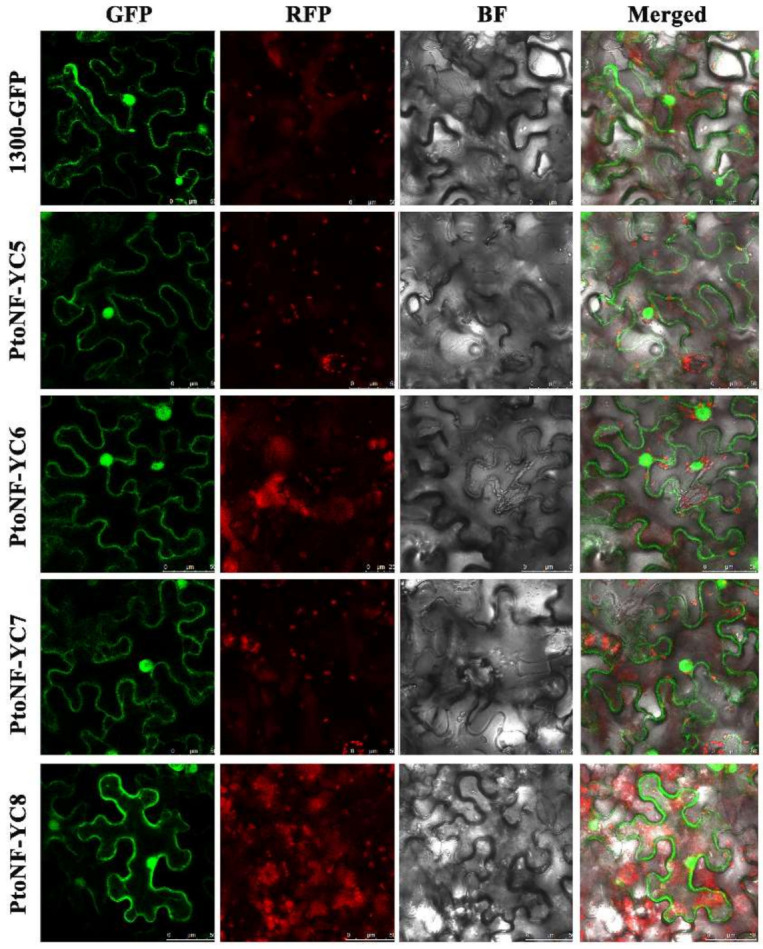
Subcellular localization of pSuper1300-GFP-PtoNF-YC5/6/7/8 proteins. From left to right, GFP, RFP, BF, and Merged represent the GFP signal, chloroplast spontaneous signal, bright field, and superimposed signal, respectively, bar 50 μm.

The function of a gene is related not only to its expression pattern but also to its location. Therefore, cellular localization has important significance for the study of proteins with unknown functions. To explore the location of the PtoNF-YC5/6/7/8 proteins, we fused the four proteins of PtoNF-YC with the GFP protein and transformed them into *Agrobacterium* GV3101 (Figure S3). The *Agrobacterium* solution of pSuper1300-GFP vector and pSuper1300-GFP-PtoNF-YC5/6/7/8 vector were injected into tobacco leaves and expressed transiently, and localization was analyzed by fluorescence. All four proteins were located in the cell membrane and nucleus. Compared with the pSuper1300-GFP vector, pSuper1300-GFP-PtoNF-YC8 exhibited a stronger fluorescence signal on the cell membrane, while the fluorescence signals of the other three proteins did not differ significantly from that of pSuper1300-GFP (Figure 3).

### 3.3. Effect of Ectopic Expression of PtoNF-YC6/8 on Early Flowering in Transgenic Arabidopsis

The sequence analysis showed that the homology of PtoNF-YC5 and PtoNF-YC6 reached 85.39%, and the homology of PtoNF-YC7 and PtoNF-YC8 was 83.53%. Compared with PtoNF-Y5 and PtoNF-YC7, PtoNF-YC6 and PtoNF-YC8 had higher similarity with AtNF-YC3 and AtNF-YC9 (Table S2). Therefore, PtoNF-YC6 and PtoNF-YC8 were selected for further research.

**Figure 4:**
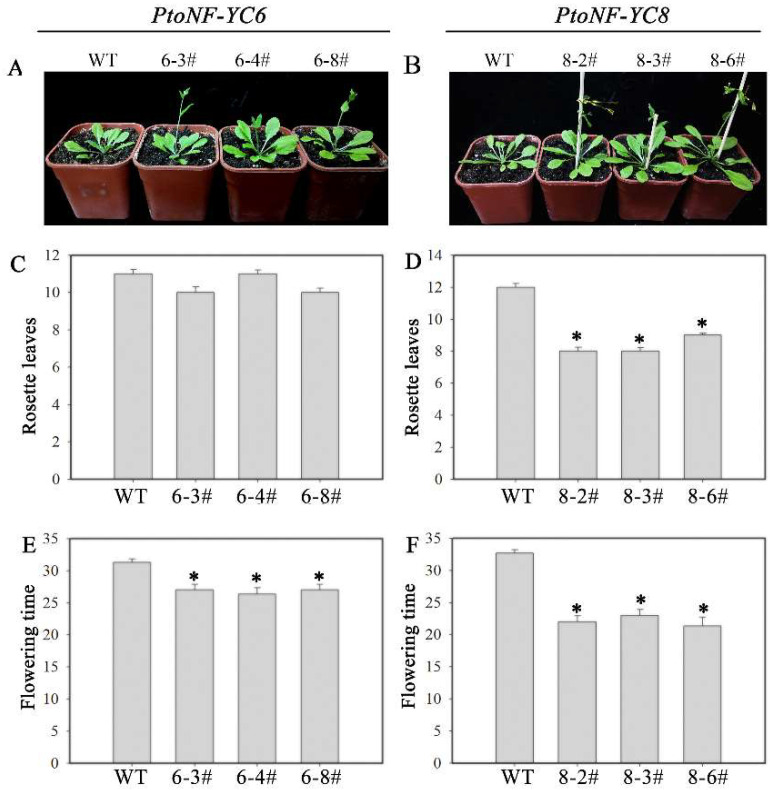
Flowering time and phenotype analysis of *PtoNF*-*YC6*/*8* transgenic *Arabidopsis*. (**A**,**B**) Images of flowering phenotype. (**C**,**D**) Analysis of rosette leaf number. (**E**,**F**) Analysis of flowering time. * (*p* < 0.05) indicate significant differences.

In this study, to determine the effects of the *PtoNF-YC6* and *PtoNF-YC8* genes on flowering time, we obtained 10 lines of *PtoNF-YC6* and 9 lines of *PtoNF-YC8* transgenic *Arabidopsis* through multi-level identification. The positive plants were continued to be cultivated and the seeds were collected from a single plant, screened again, and the seeds of the T3 generation were collected for subsequent experiments. The T3 generation plants were grown under long-day conditions (16 h light/8 h dark) and their phenotypes were observed and counted. The analysis of transcriptional expression levels showed that 6–3#, 6–4#, 6–8#, 8–2#, 8–3#, and 8–6# had the highest expression levels (Figures S4 and S5), and their flowering time were most significantly earlier than the WT. In addition, the number of rosette leaves showed differences. The results showed that the expression of *PtoNF-YC6* and *PtoNF-YC8* significantly shortened the flowering time of transgenic *Arabidopsis*, and *PtoNF-YC8* had a stronger effect than *PtoNF-YC6* (Figure 4).

### 3.4. Analysis of the Effects of PtoNF-YC6 and PtoNF-YC8 on the Floral Organs of Arabidopsis

**Figure 5:**
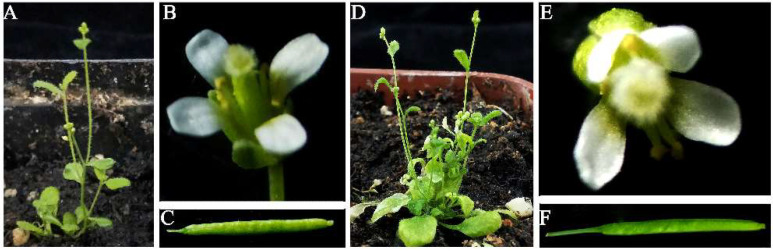
Phenotypes of floral organs in *PtoNF-YC6* transgenic *Arabidopsis*. (**A**) WT plants. (**B**) WT petals. (**C**) WT seeds. (**D**) *PtoNF-YC6* gene transgenic plants. (**E**) *PtoNF-YC6* transgenic plant petals. (**F**) *PtoNF-YC6* transgenic plant seeds.

**Figure 6:**
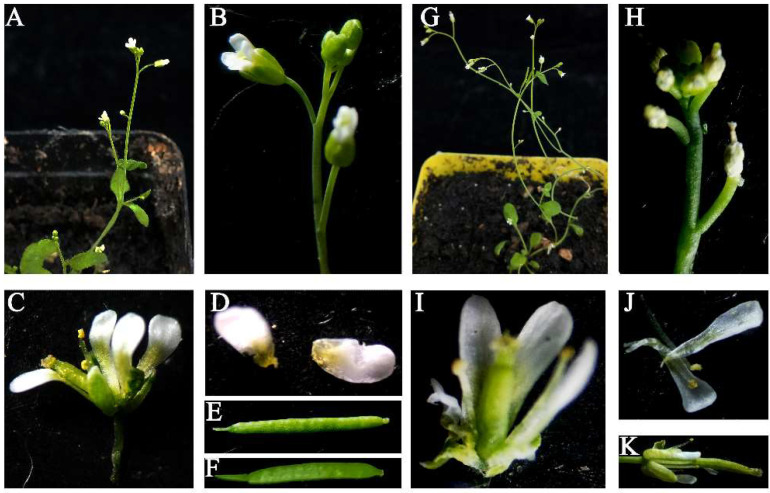
Phenotypes of floral organs in *PtoNF-YC8* transgenic *Arabidopsis*. (**A**,**G**) WT and *PtoNF-YC8* transgenic *Arabidopsis* plants, respectively. (**B**–**D**). WT plant floral morphology. (**H**–**K**) *PtoNF-YC8* transgenic *Arabidopsis* floral morphology. (**E**) WT plant seeds. (**F**) *PtoNF-YC8* transgenic plant seeds.

Next, we determined whether the *PtoNF-YC6* and *PtoNF-YC8* genes affected the morphology of *Arabidopsis* floral organs. Compared with WT plants, 6–3# transgenic plants had significantly more branches, but there was no difference in flower morphology between WT and transgenic *Arabidopsis* (Figure 5D). They both had four petals, and the petals and calyx fell off as the pod grew (Figure 5C,F). For *PtoNF-YC8* transgenic *Arabidopsis* plants, the secondary stems of line 8–6# significantly increased (Figure 6A,B,G). The *PtoNF-YC8* transgenic plants also showed a reduction in the number of petals and elongated petals (Figure 6I–K). The petals and calyx also fell off with pod growth (Figure 6E,F). In addition, some secondary branches showed floral organ abortion (Figure 6H). Overall, the results indicate that *PtoNF-YC8* plays an important role in regulating flower morphology and floral organ development.

### 3.5. Effects of PtoNF-YC6 and PtoNF-YC8 on the Growth of P. tomentosa

**Figure 7:**
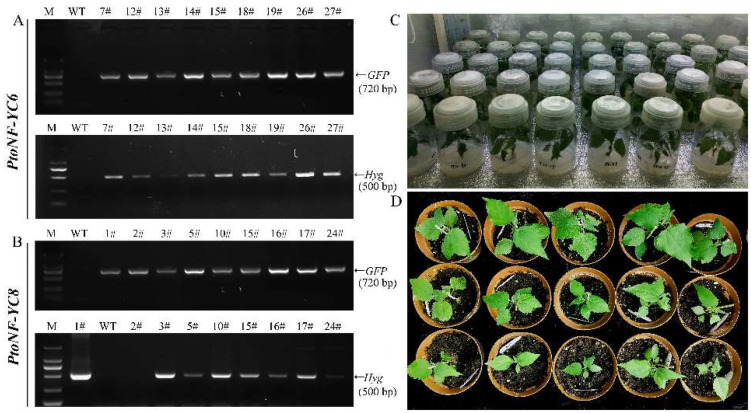
PCR identification and propagation of *PtoNF-YC6* and *PtoNF-YC8* transgenic *P. tomentosa*. (**A**) Identification of *PtoNF-YC6* transgenic lines. (**B**) Identification of *PtoNF-YC8* transgenic lines. (**C**) Transgenic plant propagation. (**D**) The transgenic plants transplanted into soil in greenhouse.

**Figure 8:**
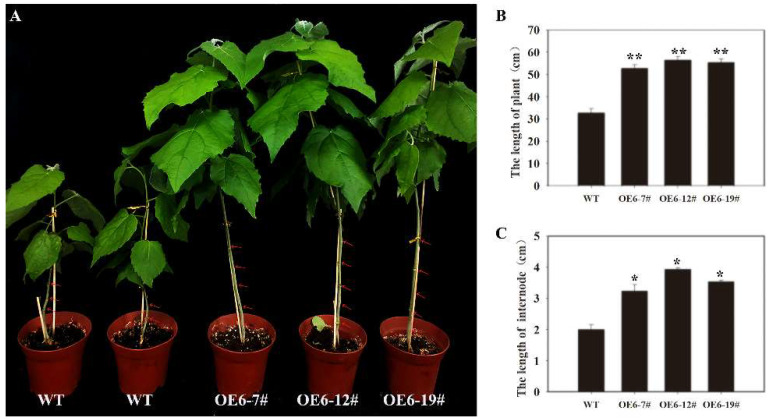
The phenotype analysis of *PtoNF-YC6* transgenic *P.* *tomentosa*. (**A**) *PtoNF-YC6* transgenic *P. tomentosa* plants with different growth phenotype. (**B**) Plant height analysis of *PtoNF-YC6* transgenic *P. tomentosa* plants. (**C**) Internode statistical analysis of *PtoNF-YC6* transgenic *P. tomentosa* plants. * (*p* < 0.05) and ** (*p* < 0.01) indicate significant differences. Subfigure **A** red arrows represent internodes.

**Figure 9:**
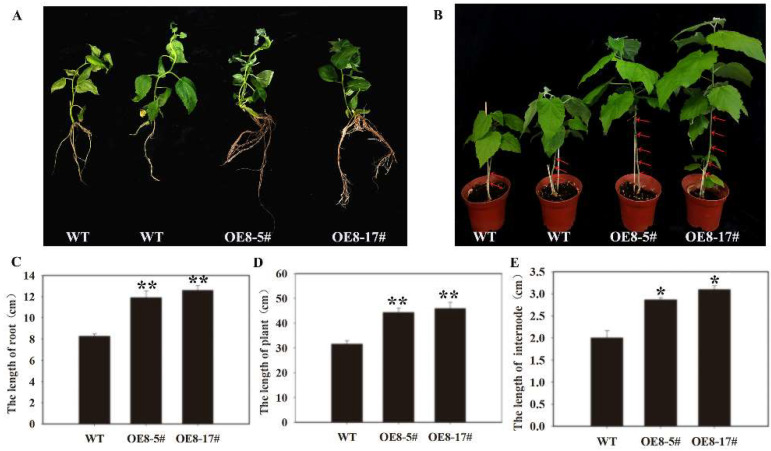
The phenotype analysis of *PtoNF-YC8* transgenic *P. tomentosa*. (**A**) The phenotypic traits of *PtoNF-YC8* transgenic *P.* *tomentosa*. (**B**) *PtoNF-YC8* transgenic *P. tomentosa* plants with different growth phenotype. (**C**) Root length analysis. (**D**) Plant height analysis. (**E**) Internode statistical analysis. * (*p* < 0.05) and ** (*p* < 0.01) indicate significant differences. Subfigure **B** red arrows represent internodes.

To verify the functions of *PtoNF-YC6* and *PtoNF-YC8* in promoting early flowering, we also obtained 12 lines of *PtoNF-YC6* and 8 lines of *PtoNF-YC8* transgenic *P. tomentosa* by PCR identification, respectively, and they were propagated and cultured for subsequent experiments (Figure 7). Subsequently, the expression levels of the transgenic plants were analyzed by RT-qPCR, the results showed that the expression levels of *PtoNF-YC6* and *PtoNF-YC8* transgenic *P. tomentosa* lines were generally 1.5–5.3 times higher than the wild-type plants, and the five plants (6–7#, 6–12#, 6–19#, 8–5# and 8–17#)with the highest expression levels were selected for subsequent phenotypic experiments (Figures S6A and S7A). In the same growth environment, we analyzed the plants grown for 6 months, and the results showed that the growth rate of the *PtNF-YC6*/*8*-overexpressing *P. tomentosa* lines was significantly faster than that of the wild-type (WT) plants. For example, when the height of wild-type plants was 33 cm, the height of *PtoNF-YC6* and *PtoNF-YC8* transgenic poplar was 45–55 cm. The height of the plant mainly depends on the internode distance of the plant. Therefore, the internode distance was measured and counted. The results showed that the internode distance of the transgenic plant was significantly larger than that of the wild type. However, the plants obtained so far do not show early flowering, and further cultivation and observation are needed to determine their function (Figure 8 and Figure 9).

### 3.6. The Possible Molecular Mechanism of PtoNF-YC6/8 Expression in P. tomentosa Promoting Early Flowering

**Figure 10:**
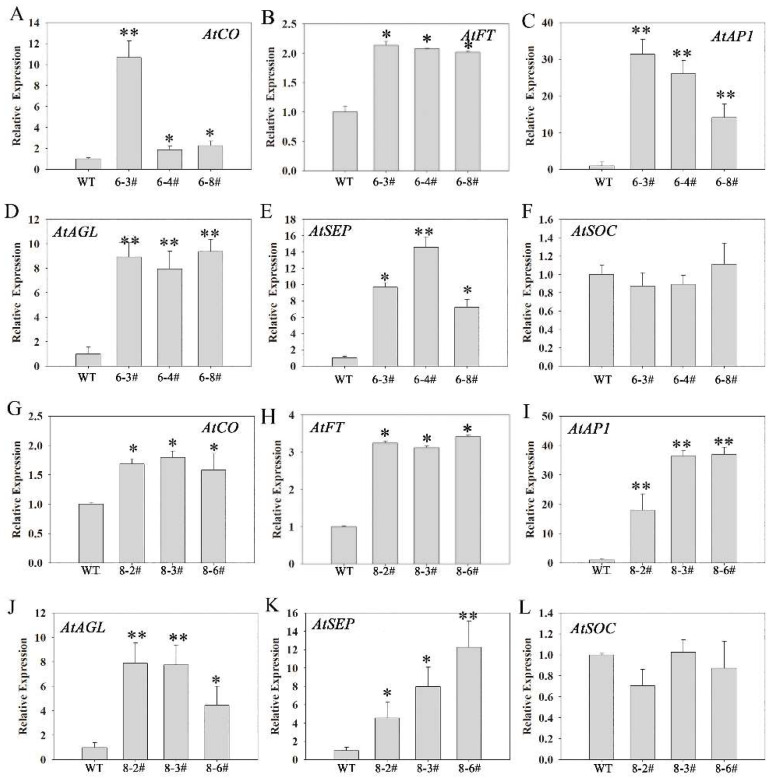
Analysis of the expression patterns of flowering time-related genes in regulated *PtoNF-YC6* and *PtoNF-YC8* transgenic *Arabidopsis*. (**A**–**F**) represent the expression levels of *AtCO, AtFT, AtAP1, AtAGL, AtSEP* and *AtSOC* in *PtNF-YC6* transgenic *Arabidopsis*, respectively. (**G**–**L**) represent the expression levels of *AtCO, AtFT, AtAP1, AtAGL, AtSEP* and *AtSOC* in *PtNF-YC8* transgenic *Arabidopsis*, respectively. * (*p* < 0.05) and ** (*p* < 0.01) indicate significant differences.

To further analyze the mechanism of how *PtoNF-YC6* and *PtoNF-YC8* regulate plant early flowering, we used the leaves of WT (Col), *PtoNF-YC6,* and *PtoNF-YC8* transgenic plants before bolting as samples and analyzed the expression levels of flowering genes by qRT-PCR. In *PtoNF-YC6* transgenic *Arabidopsis* lines, except for *AtSOC*, the expression levels of *AtCO*, *AtFT*, *AtAP*, *AtAGL*, and *AtSEP3* were higher than those of the WT (Figure 10A–F). For *PtoNF-YC8* transgenic plants, the expression pattern of flowering-related genes was similar to that of *PtoNF-YC6* transgenic plants (Figure 10G–L). Thus, we speculate that overexpression of *PtoNF-YC6* and *PtoNF-YC8* genes can upregulate the expression level of *AtCO*, and then activate the downstream expression of flowering genes such as *AtFT*, and ultimately advance flowering in *Arabidopsis*. Meanwhile, we also analyzed the expression levels of flowering-related genes such as *PtoCO*, *PtoFT*, *PtoSOC*, *PtoAP1,* and *PtoAGL* by qRT-PCR. The analysis results showed that *PtoNF-YC6* and *PtoNF-YC8* were similar, and *PtoCO*, *PtoFT*, *PtoAP*1, and *PtoAGL* were generally up-regulated in transgenic *P. tomentosa* lines, especially 6–7#, 6–12#, 6–19#, and 8–17# (Figures S6B–F and S7B–F).

**Figure 11:**
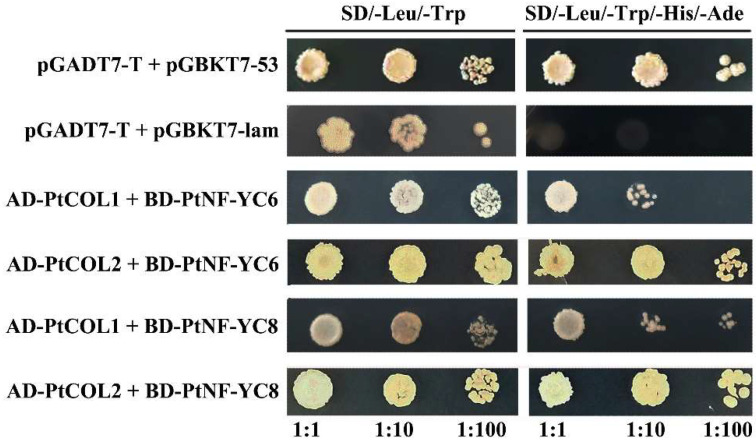
Y2H analysis of the interactions between PtoNF-YCs and PtoCOLs; 1:1, 1:10, and 1:100 represent the interaction of undiluted bacterial solution, 10-fold dilution and 100-fold dilution, respectively.

CO plays an important role in photoperiod regulation of the flowering pathway of *Arabidopsis* [40]. Studies have shown that *PtoCOL1* and *PtoCOL2* have similar expression patterns to *AtCOL1*, *AtCOL2*, and *AtCO* in *Arabidopsis*. Meanwhile, the genetic relationship also showed that PtCOL1/2 and AtCOL1, AtCOL2, and AtCO protein form a branch [41]. Therefore, PtoCOL1/2 may have similar functions to AtCOL1, AtCOL2, and AtCO. We further used the Y2H system to verify the interaction between PtoNF-YC6/8 and PtoCO1/CO2 proteins. The results indicated that pGADT7-PtoCOL1 and pGBKT7-PtoNF-YC6/8, as well as the combination of pGADT7-PtoCOL2 and pGBKT7-PtoNF-YC6/8, can grow normally on SD-Trp-Leu-His-Ade medium (Figure 11). Thus, we speculate that *PtoNF-YC6* and *PtoNF-YC8* can promote flowering by interacting with PtoCOL1 and PtoCOL2 proteins.

## 4. Discussion

The *NF-Y* gene has been isolated and analyzed in a variety of plants. We previously identified the poplar NF-Y gene family through bioinformatics methods, and initially screened four *PtNF-YC* genes related to flowering [16]. In this study, four *PtoNF-YC* genes were cloned from *P. tomentosa* TC1521. The multiple alignment analysis showed that all four members of PtoNF-YC have three α-helix structures and αC structures, and are highly conserved with AtNF-YC3 and AtNF-YC9 proteins (Figure 1A,B). In addition, the phylogenetic analysis showed that all NF-YC proteins were divided into two groups, while the four members of PtoNF-YC formed a branch with dicot plants and were more closely related to cotton and alfalfa (Figure 1C). Phylogenetic analysis showed that after the differentiation of angiosperms into monocots and dicots plants, many gene families have undergone gene duplication events [42], resulting in various differences in many aspects such as flower formation and flowering time. Although research has investigated the floral organs and flowering time of dicot plants, little is known about the regulation mechanism of flower development in poplar, an important dicot plant and woody plant model. Therefore, we studied the function of the *PtoNF-YC* gene of *P. tomentosa*, providing an important theoretical basis for improving the molecular regulation of flowering in dicots and woody plants.

The subcellular localization of the *NF-Y* gene has been reported in many species, and has indicated that the protein is mainly localized in the nucleus. For instance, nuclear localization has been observed for the PdNF-YB protein [43] and the PtNF-YA6 and PtNF-YA9 proteins of poplar [29]. However, NF-Y proteins are also localized elsewhere. For example, the Cdt-NF-YC1 fusion protein is localized in the nucleus and the periphery of the cell [43], and the TaNF-YA10 protein of wheat is localized in the nucleus and cytoplasm [44]. Recent studies have shown that poplar NF-YA3 is localized in the nucleus and cell membrane under normal conditions, but when exposed to external stress, NF-YA3 mainly accumulates in the nucleus [45]. In this study, the four pSuper1300-GFP-PtoNF-YC5/6/7/8 proteins were localized in the nucleus and cell membrane (Figure 3). However, in *PtoNF-YC6*/*8* transgenic *Arabidopsis*, the fluorescence signal was only located in the nucleus and guard cells (Figure S5). In addition, the CO protein is localized in the nucleus. Thus, we speculate that PtoNF-YC6/8 mainly accumulates in the cell nucleus and interacts with CO protein to induce the expression of downstream flowering genes and regulate flowering.

To further study the role of *PtoNF-YC* in flowering regulation, we genetically transformed the *PtoNF-YC6* and *PtoNF-YC8* genes. Because poplar has a long juvenile period and *Arabidopsis* as a model plant has the characteristics of a short lifecycle and mature genetic transformation system, there is a need to study the gene function of species with a long juvenile period. The two genes *PtoNF-YC6* and *PtoNF-YC8* were transformed into *Arabidopsis*, and their phenotypes were observed and counted. We observed that the transgenic *Arabidopsis* with *PtoNF-YC6* and *PtoNF-YC8* showed early flowering (Figure 4), consistent with the expected results. AtNF-YC3/4/9 and AtNF-YB2/3 first form a dimer, and then complex with CO in the nucleus, thereby promoting *Arabidopsis* early flowering [22]. In addition, AtNF-YC2, the first AtNF-YC member isolated from plants, can upregulate *FT* expression, thereby promoting early flowering in *Arabidopsis* [46].Tomato HAP5a can also trigger early flowering in *Arabidopsis* [47]. Moreover, the five members of *TaNF-YC5*/*8*/*9*/*11*/*12* in wheat are regulated by light signals to participate in the regulation of flowering time [48]. In general, homologous genes usually have the same or similar functions. Here, we further analyzed the expression levels of genes related to the flowering pathway of *PtoNF-YC6* and *PtoNF-YC8* transgenic *Arabidopsis* plants, and observed significantly upregulated expression levels of *AtCO*, *AtFT*, *AtAP1*, *AtAGL*, and other related genes (Figure 10). This suggests that *PtoNF-YC6* and *PtoNF-YC8* not only interact with CO to promote target gene expression, but also promote CO expression itself in *A. thaliana*. Therefore, it is further speculated that PtoNF-YC6 and PtoNF-YC8 have similar regulatory mechanisms to AtNF-YC3, AtNF-YC4, and AtNF-YC9 in flowering regulation. In addition, studies have shown that in *Arabidopsis*, NF-Y can regulate plant flowering through aging and gibberellic acid pathways [23,28], but whether poplar NF-Y members play a role in these pathways needs to be further explored.

**Figure 12:**
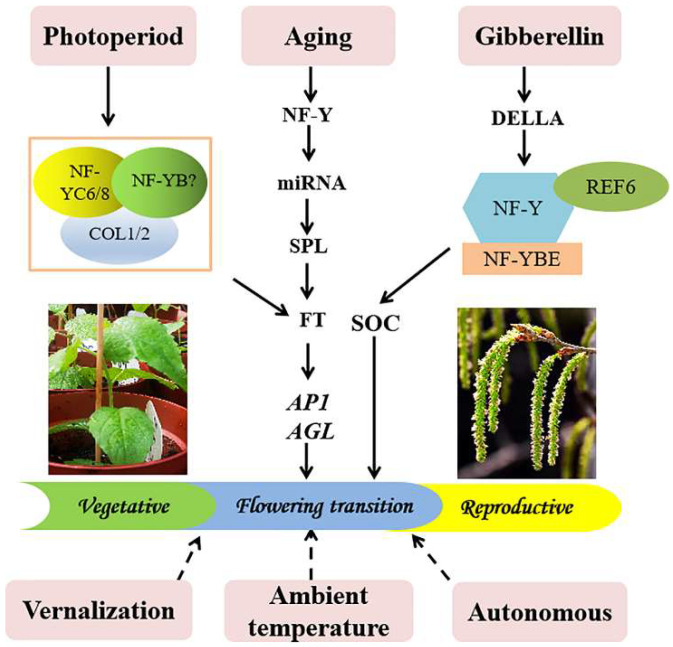
Flowering time network regulation by NF-Y.

Poplar has been regarded as the model plant among woody plants. As an important native tree species in China, *P. tomentosa* plays an important role in rural greening and ecological protection. *P. tomentosa* has a long juvenile phase, which seriously affects the poplar breeding cycle. Therefore, it is very important to choose *P. tomentosa* as the research object to study the molecular mechanism of its flowering regulation, and it also provides a reference value for the flowering regulation of other woody plants. In this study, transgenic *PtoNF-YC6* and *PtoNF-YC8* plants were also obtained, and it was observed that transgenic *P. tomentosa* plants grew better than WT plants, which may have caused the transgenic plants to enter the reproductive growth stage earlier. CO, as an important transcription factor in the photoperiod response pathway, is usually up-regulated under long-day conditions, further promoting the expression of FT and activating the key gene *AP1* in floral meristems, and finally making plants flower early [40]. In this study, the expression level of *PtoCO* gene was up-regulated 2–7 times in both *PtoNF-YC6* and *PtoNF-YC8* transgenic lines, and key flowering integrons such as *PtoFT* and *PtoAGL* and key genes of floral meristem in transgenic lines are also up-regulated (Figures S6 and S7), indicating that this will have an impact on the flowering time and flowering development of *P. tomentosa*. In addition, Y2H experiments also showed that PtoCOL1 and PtoCOL2 proteins can interact with PtoNF-YC6 and PtoNF-YC8 proteins (Figure 11). Research showed that PtoCOL1 and PtoCOL2 have similar expression patterns and are closely related to AtCOL1, AtCOL2, and AtCO in *Arabidopsis* [41,49]. Therefore, we further speculated that PtoNF-YC6 and PtoNF-YC8 are similar to AtNF-YC3, AtNF-YC4, and AtNF-YC9 in flowering regulation. The regulation mechanism also involves interactions with the CO protein, which then regulates the expression of downstream flowering genes, and ultimately promotes flowering (Figure 12). However, the obtained transgenic *P. tomentosa* plants did not show the expected early flowering (Figure 8 and Figure 9). There are three possible reasons for this. First, NF-Y transcription factors are important regulators of epigenetic marks that control flowering. Second, as a perennial woody plant, poplar has a long juvenile period. Third, NF-Y usually functions in a trimeric form. Therefore, whether PtoNF-YA and PtoNF-YB are involved in the formation of the PtoNF-Y complex that regulates poplar flowering requires further analysis through epigenetics and proteomics [50,51]. In addition, whether the polymer formed by the PtoNF-YC protein of poplar is bound to the promoter region of key flowering genes requires further analysis to clarify the downstream genes and specific mechanisms regulated by *PtoNF-YC*.

## 5. Conclusions

In this study, four *PtoNF-YC* genes were cloned from *P. tomentosa*, and their characterization and function were analyzed. Overall, our results showed that although these genes are highly similar in sequence, there are some differences in their functions. In addition, the mechanism of *PtoNF-YC6* and *PtoNF-YC8* involved in plant flowering regulation was analyzed, laying a theoretical foundation for follow-up studies on the molecular regulation network of poplar *NF-Y* regulating flowering, and providing a reference for research on the flowering regulation of other woody plants.

## Data Availability

Not applicable.

## Supplementary Materials

The following supporting information can be downloaded at: https://www.mdpi.com/article/10.3390/ijms23063116/s1.
