# Supplementary material for: Comprehensive Analyses of Four PtoNF-YC Genes from Populus tomentosa and Impacts on Flowering Timing
Source: Int J Mol Sci. 2022 Mar 14;23(6):3116. doi: 10.3390/ijms23063116 (PMC8950544; doi:10.3390/ijms23063116)
Supplement: Supplementary file 1 [file ijms-23-03116-s001.zip › ijms-1573529 - supplementary.pdf]

Table S1. Primer sequences of this study.

Name	Sequence（5′-3′）	Usage
PtoNF-YC5-F	GC**GTCGAC**ATGGAGCAAGG	Cloning
PtoNF-YC5-R	GG**ACTAGT**CTGAGGGCGACTG	
PtoNF-YC6-F	GC**GTCGAC**ATGGATCAGCAAG	
PtoNF-YC6-R	GG**ACTAGT**CTACTGATCTGAGGGTGAC	
PtoNF-YC7-F	GC**GTCGAC**ATGATGGGACCTT	
PtoNF-YC7-R	GG**ACTAGT**TCAGGAATCTTCAGGTGGC	
PtoNF-YC8-F	GC**GTCGAC**ATGGACCAGCA	
PtoNF-YC8-R	GG**ACTAGT**TCAGGAATCTTCAGGTGGCT	
PtoNF-YC5-F	CGGTCTTGGAATCACACGGA	qRT-PCR
PtoNF-YC5-R	CCGACAGGCATTGTTCCTCT	
PtoNF-YC6-F	GAACAATGCCTGTTGGAGGG	
PtoNF-YC6-R	TGTCACAGGCTTGCCCATTA	
PtoNF-YC7-F	AGTCCCGAGAGGGAGTCTTC	
PtoNF-YC7-R	TTTGCTGTGGCCAAATCTGC	
PtoNF-YC8-F	GGAAAGCCTGTGGTGGATCA	
PtoNF-YC8-R	AGGAATCTTCAGGTGGCTGC	
AtCO-F	CAGGCAAACAGTGTATGCACCAGG	
AtCO-R	CCGCAGCCACTCTCCCTCTG	
AtSOC-F	GGGATCTCATGAAAGCGAAGTTTG	
AtSOC-R	CTTGAAGAACAAGGTAACCCAATGAA	
AtSVP-F	GGAAAACTGTTCGAGTTCTG	
AtSVP-R	TCTTTGTTTCAATCACACGC	
AtFT-F	AGAAGACTTTAGATGGCTTCTT	
AtFT-R	TTATCGCATCACACACTATATAAG	
AGL-F	GGCTGGAGAAACTACTTGAA	
AGL-R	TTTAAGCGTCGTCAGTTTTG	
AP1-F	CATGGGTGGTCTGTATCAAGAAGAT	
AP1-R	CATGCGGCGAAGCAGCCAAGGTT	
AtSEP3-F	CGGTCGTCATCATCATCAACA	
AtSEP3-R	GTCCTGCTCCCATTCCATCTT	
PtACTIN-F	CTCCATCATGAAATGCGATG	
PtACTIN-R	TTGGGGCTAGTGCTGAGATT	

AtUBQ-F	GGGCACTCAAGTATCTTGTTAGC	
AtUBQ-R	TGCTGCCCAACATCAGGTT	
AD-PtCOL1-F	CG**GAATTC**ATGCCACGTGTCAC	
AD-PtCOL1-R	CG**GGATCC**GAATGATGGGACAAT	
AD-PtCOL2-F	CG**GAATTC**ATGTTGAAGCAAGAGAGTAGTGG	
AD-PtCOL2-R	CG**GGATCC**GAATGATGGGACAAT	Y2H vector construction
BD-PtNF-YC6-F	CG**GAATTC**ATGGATCAGCAAGGGTATGGG	
BD-PtNF-YC6-R	CG**GGATCC**CTGATCTGAGGGTGACTGCTG	
BD-PtNF-YC8-F	CG**GAATTC**ATGGACCAGCAAGGCCATG	
BD-PtNF-YC8-R	CG**GGATCC**GGAATCTTCAGGTGGCTGCT	

Table S2. Comparison of similarity between PtoNF-YC5/6/7/8 protein and AtNF-YC3/9 protein.

Gene Members	PtoNF-YC5	PtoNF-YC6	PtoNF-YC7	PtoNF-YC8
**AtNF-YC3**	43.97%	55.00%	32.39%	46.51%
**AtNF-YC9**	51.74%	58.44%	36.06%	53.10%

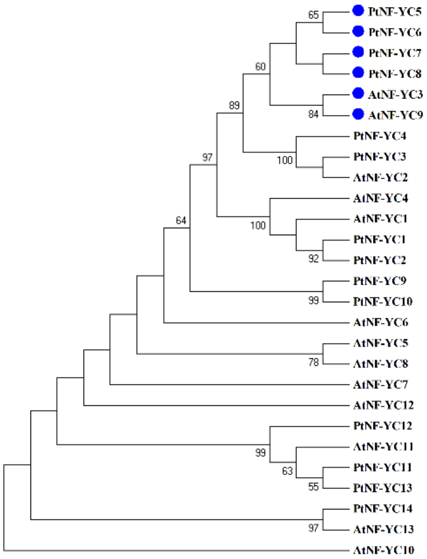

Figure S1. Phylogenetic trees constructed by NF-Y proteins from Arabidopsis and *Populus*.

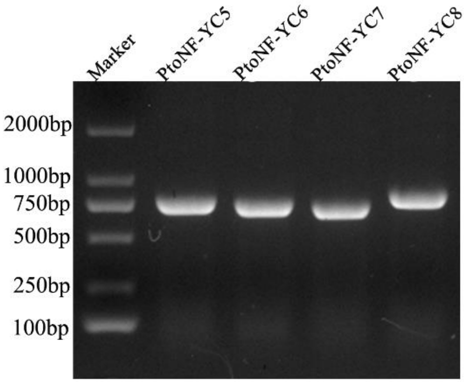

Figure S2. Amplification of the CDS of *PtoNF-YC5/6/7/8*.

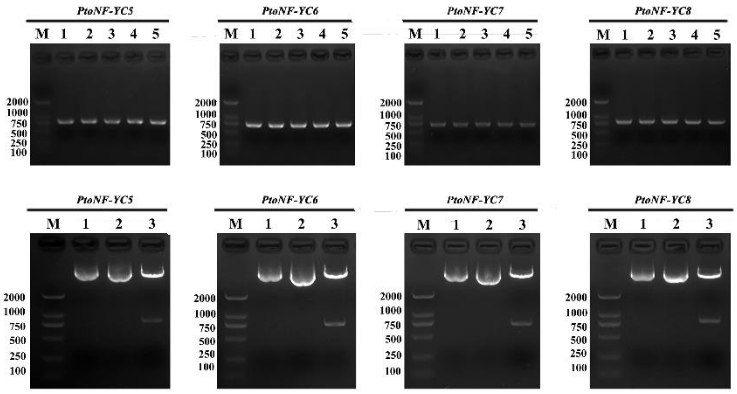

Figure S3. pSuper1300-GFP-PtoNF-YC5/6/7/8 bacterial liquid PCR and plasmid double enzyme digestion identification. M: 2000 DNA Maker, (below) 1: pSuper1300-GFP empty vector, 2: pSuper1300-GFP-PtoNF-YC5/6/7/8 vector, 3: pSuper1300-GFP-PtoNF-YC5/6/7/8 plasmid double restriction digestion identification.

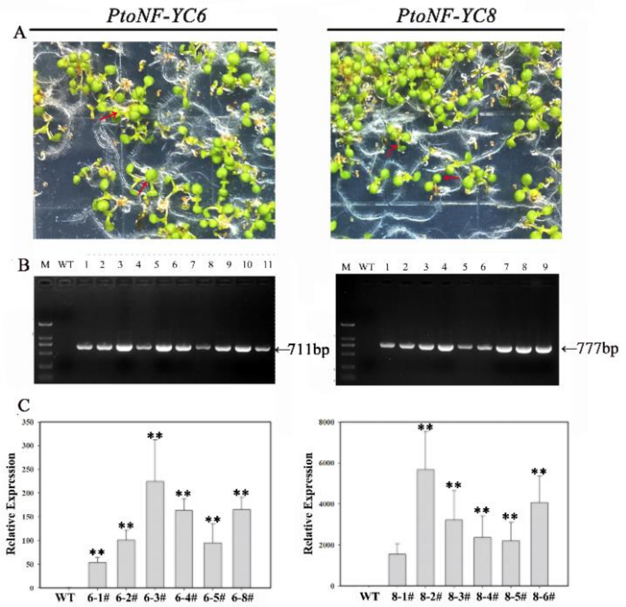

Figure S4. Identification of *PtoNF-YC6* and *PtoNF-YC8* transgenic Arabidopsis. (**A**) Hygromycin screening of transgenic Arabidopsis. (**B**) DNA level identification the transgenic Arabidopsis. (**C**) Transcription level analysis. ** $p < 0.01$.

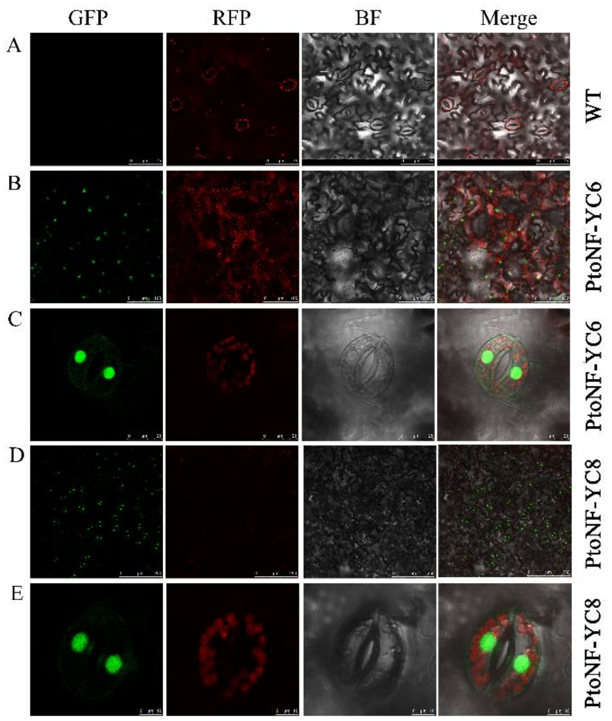

Figure S5. *PtoNF-YC6* and *PtoNF-YC8* visualized under fluorescence microscopy in transgenic Arabidopsis. (**A**) Leaf fluorescence signal of wild-type seedlings. (**B**,**D**). Leaf fluorescence signal of transgenic *PtoNF-YC6* and *PtoNF-YC8* plants, bar 100μm. (**C**,**E**). Fluorescence signal of *PtoNF-YC6* and *PtoNF-YC8* transgenic leaf guard cells, bar 25 μm.

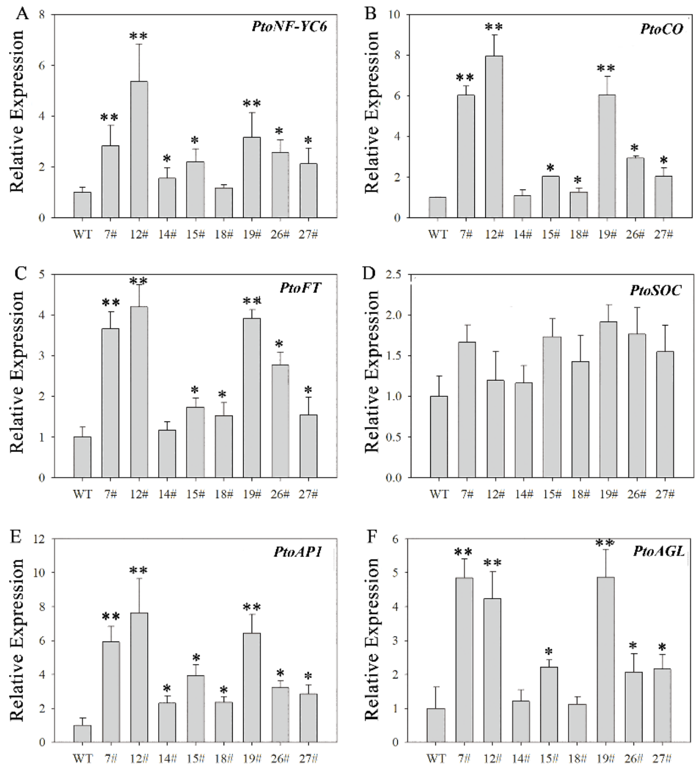

Figure S6. Expression analysis of *PtoNF-YC6* and endogenous flowering genes of *P. tomentosa* (**A–F**). * $p < 0.05$, ** $p < 0.01$.

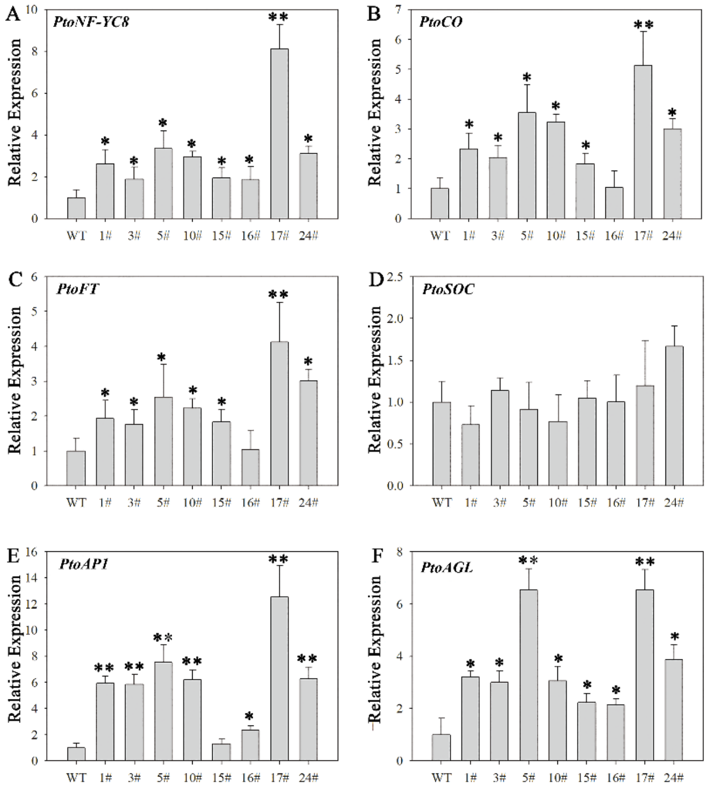

Figure S7. Expression analysis of *PtoNF-YC8* and endogenous flowering genes of *P. tomentosa* (**A**–**F**). * $p < 0.05$, ** $p < 0.01$.
